# Analysis of Social Determinants and the Utilization of Pediatric Tele–Urgent Care During the COVID-19 Pandemic: Cross-sectional Study

**DOI:** 10.2196/25873

**Published:** 2021-08-30

**Authors:** Saif Khairat, Phillip McDaniel, Matthew Jansen, Tia Francis, Barbara Edson, Robert Gianforcaro

**Affiliations:** 1 Cecil G. Sheps Center for Health Services Research Chapel Hill, NC United States; 2 Carolina Health Informatics Program University of North Carolina at Chapel Hill Chapel Hill, NC United States; 3 School of Nursing University of North Carolina at Chapel Hill Chapel Hill, NC United States; 4 Digital Research Services Department University of North Carolina at Chapel Hill Chapel Hill, NC United States; 5 UNC Health Morrisville, NC United States

**Keywords:** telehealth, pediatrics, social, determinants, COVID-19, use, children, infant, consultation, telemedicine, urgent care, vulnerable population, cross-sectional, minority

## Abstract

**Background:**

Telehealth is increasingly used to provide specialty consultations to infants and children receiving care. However, there is uncertainty if the COVID-19 pandemic has influenced the use of telehealth among vulnerable populations.

**Objective:**

This research aims to compare the overall use of tele–urgent care visits for pediatric patients before and after the pandemic, especially among vulnerable populations.

**Methods:**

We conducted a cross-sectional analysis of pediatric tele–urgent care visits at a virtual care center at a southeastern health care center. The main outcome of this study was the use of pediatrics tele–urgent visits across geographical regions with different levels of social disparities and between 2019 and 2020.

**Results:**

Of 584 tele–urgent care visits, 388 (66.4%) visits occurred in 2020 during the pandemic compared to 196 (33.6%) visits in 2019. Among 808 North Carolina zip codes, 181 (22%) consisted of a high concentration of vulnerable populations, where 17.7% (56/317) of the tele–urgent care visits originated from. The majority (215/317, 67.8%) of tele–urgent care visits originated from zip codes with a low concentration of vulnerable populations. There was a significant association between the rate of COVID-19 cases and the concentration level of social factors in a given Zip Code Tabulation Area.

**Conclusions:**

The use of tele–urgent care visits for pediatric care doubled during the COVID-19 pandemic. The majority of the tele–urgent care visits after COVID-19 originated from regions where there is a low presence of vulnerable populations. In addition, our geospatial analysis found that geographic regions with a high concentration of vulnerable populations had a significantly higher rate of COVID-19–confirmed cases and deaths compared to regions with a low concentration of vulnerable populations.

## Introduction

Pediatricians have used telehealth to provide a broad range of health care services among primary and specialty care [1-3]. Telehealth is increasingly used to provide specialty consultations to infants and children receiving care [4]. However, since the pandemic, the use of tele–urgent care for pediatrics has grown to include telephone consultations and remote surveillance or to replace in-person primary care visits without sufficient evaluation of these interventions [5-7]. There is limited knowledge on the use of tele–urgent care to respond to urgent care needs in pediatrics during the COVID-19 pandemic.

Social determinants of health are paramount to the use of tele–urgent care because of the widening digital divide that occurs as a result of differences in individual socioeconomic characteristics [8]. Social determinants of health can be defined as the circumstances that impact the health of individuals from birth to death including socioeconomic, educational, and access to health care [9]. Prior to the COVID-19 pandemic, health disparities and inequity demonstrated differences in adoption of tele–urgent care among vulnerable populations [10,11]. Vulnerable populations are defined as populations that are at risk for health access because of economic, ethnic, or health characteristics [12]. In this study, we refer to vulnerable populations based on race, socioeconomic status, and health insurance status. There is uncertainty if the COVID-19 pandemic has influenced the use of tele–urgent care among vulnerable populations. Therefore, the objective of this study was to compare the overall use of tele–urgent care services for pediatric patients before and after the COVID-19 pandemic especially among vulnerable populations.

## Methods

### Overview

We conducted a cross-sectional analysis of pediatric tele–urgent care visits at a virtual care center at a southeastern health care center. The virtual care center offers on-demand services to all patients older than 2 years regardless of their geographic location or medical affiliation to a health care system. Patients are required to create a profile through the virtual care center web portal and provide personal information such as age, gender, residential address, and insurance coverage. The web portal allows patients to choose from a list of providers based on the patient’s chief complaint. In addition, patients can choose between having an on-demand visit or scheduling a visit based on their preference and the availability of the provider. Board-certified physicians are available for on-demand televisits with the ability to prescribe and send medications to the patient’s choice of pharmacy.

### Data and Materials

The tele–urgent care visit data were received and preprocessed in Excel (Microsoft Corporation). All patients between the ages of 2 and 18 years were included in the analysis. Gender had three categories that were male, female, and nonbinary. Insurance type included the member ID and group ID if the patient provided insurance coverage information. Otherwise, the insurance field was empty, indicating the patient reported no insurance coverage.

### Outcomes

The main outcome of this study was the use of pediatrics tele–urgent care visits across geographical regions with different levels of social disparities and between 2019 and 2020.

### Data Analysis

Since tele–urgent care participant data were collected and available at the zip level, Zip Code Tabulation Areas (ZCTAs; generalized areal representations of United States Postal Service zip code service areas) were used as the unit of analysis [13]. A variety of detailed demographic data are available at the ZCTA level from the American Community Survey (ACS) [14]. We developed social factors based on previous social determinants of health models [15], including our own model to assess health disparities in the use of tele–urgent care [10]. We collected daily COVID-19 case counts in North Carolina zip codes during the study period to assess if there was a relationship between the prevalence of COVID-19 and the use of tele–urgent care for pediatrics within North Carolina zip codes.

For this research, ZCTA-level social factors data was obtained from the ACS 2014-2018 5-year estimates, the most current 5-year data available from the ACS. We used percentages to account for population density for each social factor. Social factors used in the analyses were percent American Indian or Alaska Native people, percent of Black or African American people, percent in poverty, percent of single female headed households receiving Supplemental Nutrition Assistance Program (SNAP; formerly known as food stamps) with children younger than 18 years, percent of households receiving food stamps or SNAP with a person older than 60 years, total population receiving Medicare, and total population receiving Medicaid. For each variable, a threshold was set to determine if a ZCTA was *at risk* (ZCTAs with a value over the threshold were coded with a 1, while those below were coded with a 0). Scores across all factors were tabulated for all ZCTAs to create an aggregate risk and deprivation score (higher aggregate scores indicate greater risk and deprivation). The coding of ZCTA counts were not mutually exclusive among social factors.

The maps were created using the computed social score for each ZCTA, as well as data on 2019 and 2020 telemedicine visits, and COVID-19 cases, aggregated to ZCTAs. For reference, urban centers throughout the state are labeled on the 2019 visits map. We used dot plot graphs to represent the number of visits coming from zip codes tagged with each of the listed social factors, tagged with no social factors, and the overall visit counts for each year for reference. Factors were sorted by the overall frequency of visits across both years.

Descriptive statistics, visualizations, and statistical tests were all performed in R (R Foundation for Statistical Computing) using ggplot2, version 4.0.2., and the maps were created using ArcGIS Pro 2.6.0 (Esri; July 28, 2020) and Illustrator 2020 24.3.0 (Adobe Inc; August 1, 2020). We primarily analyzed the data through descriptive tables and visualizations.

## Results

Of 584 tele–urgent care visits, 388 (66.4%) visits occurred in 2020 during the pandemic compared to 196 (33.6%) visits in 2019. Over half of the patients were male (112/196, 57%) in 2019, versus in 2020 when over half of the patients were female (202/388, 52%). For both years, the majority of patients reported having health insurance coverage. There was a larger gap between insured and uninsured patients in 2020, such that there were 265 (68%) insured patients and 123 (32%) uninsured patients (Table 1).

**Table 1:** Patient characteristics of tele–urgent care visits between March 1 and September 30, 2019 and 2020.

Variables		2019 visits (n=196), n (%)	2020 visits (n=388), n (%)	Ratio of increase	Total (N=584), n (%)
**Gender**					
	Male	112 (57.1)	183 (47.2)	1.63	295 (50.5)
	Female	84 (42.9)	202 (52.1)	2.4	286 (49.0)
	Nonbinary	0 (0.0)	3 (0.8)	3	3 (0.5)
**Insurance coverage**					
	Insured	108 (55.1)	265 (68.3)	2.45	373 (63.9)
	Uninsured	88 (44.9)	123 (31.7)	1.39	211 (36.1)

**Figure 1:**
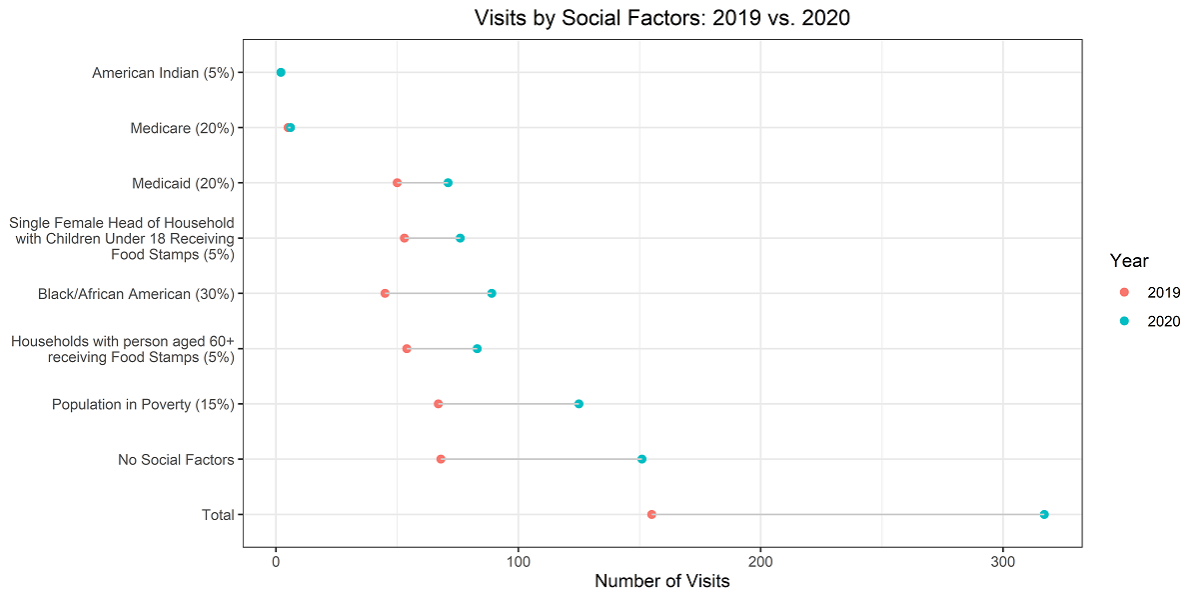
Line graph presenting pediatrics tele–urgent care visits falling under each social flag during 2019 and 2020.

The line graph in Figure 1 shows the tele–urgent care use over time broken up by social flag categories. Most of the groups show a clear increase in visits in 2020 compared to 2019. The pattern of visits over time (a peak in March followed by variable but generally lower visit counts in April through August) were not remarkably different between the time periods.

This line graph shows the percentage of the total visits across the March to August time frame within the overall telemedicine population and broken down into the zip codes with and without social factors identified. The year 2019 shows more variation around the overall trend, reflecting a year not driven by COVID-19 and the smaller overall number of visits.

**Figure 2:**
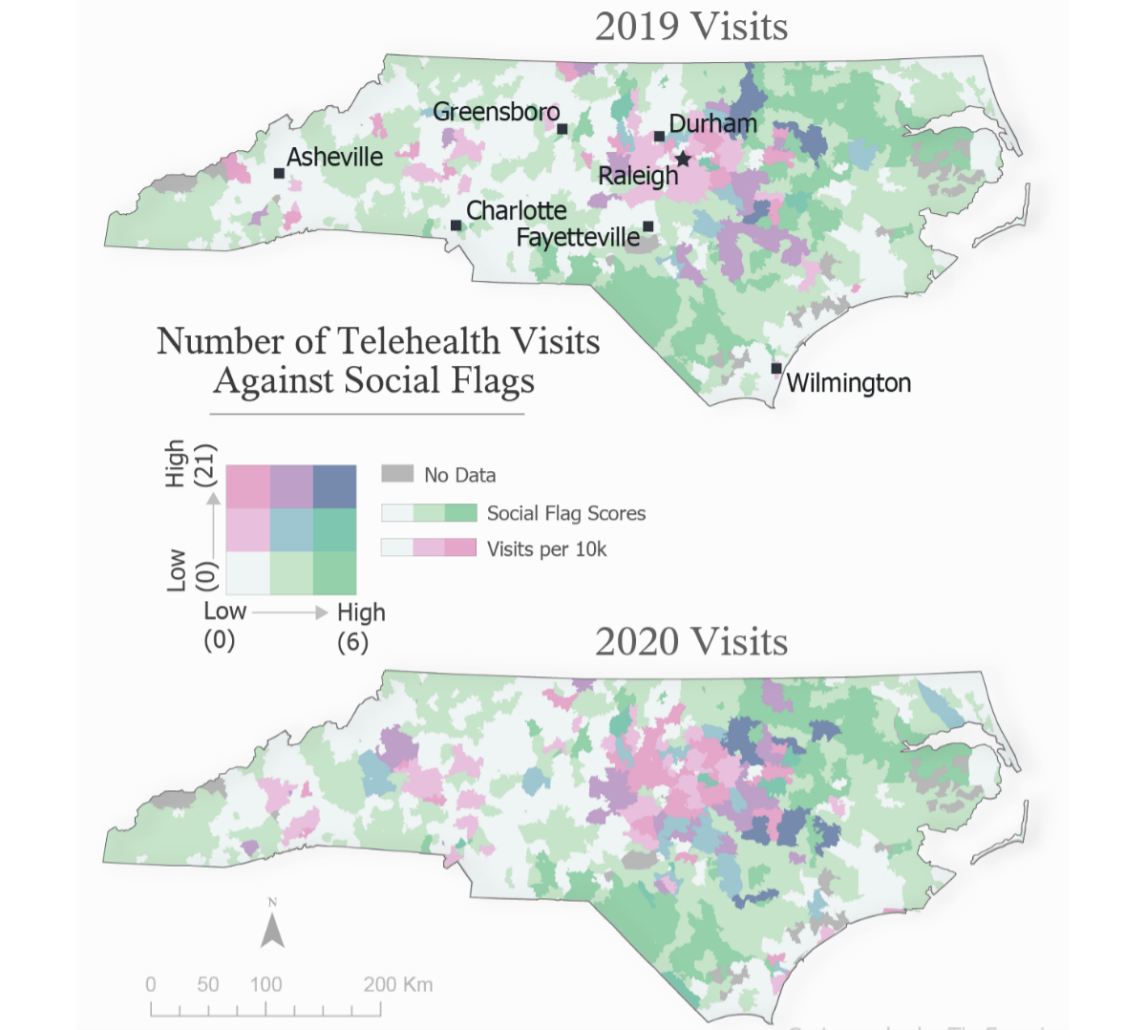
Bivariate choropleth map comparing the number of visits per 10,000 residents younger than 17 years against social flag scores. Figure produced using ArcGIS Pro 2.6.0 (July 28, 2020) and Illustrator 2020 24.3.0 (August 1, 2020).

Visits from 2019 and 2020 were also compared geographically. Figure 2 compares the number of tele–urgent care visits in each year against the social deprivation score for each zip code. Overall, there was an increase in visits in 2020, with much of the increase occurring in the central part of the state (Raleigh–Durham–Cary), an area with high population density with large medical centers. The areas colored in light gray demonstrate the ZCTAs with low social factors, while the areas colored in dark green represent areas with high social factors.

**Figure 3:**
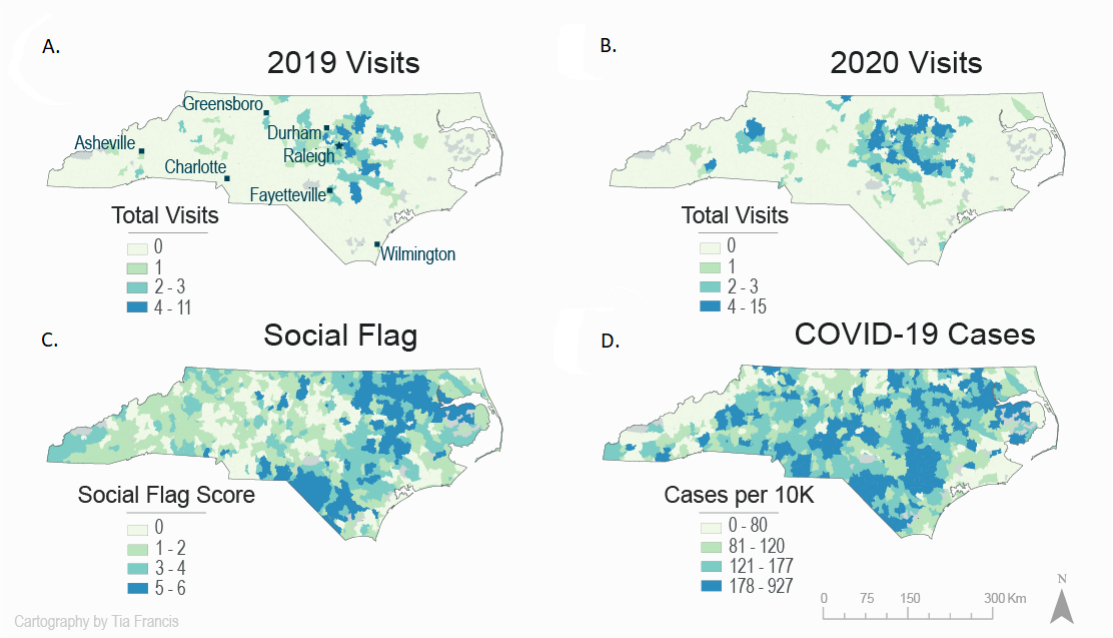
(A) Map of the tele–urgent care pediatric visits in 2019, (B) the tele–urgent care pediatric visits in 2020, (C) the categorization of North Carolina zip codes based on social vulnerability factors, (D) map of North Carolina with COVID-19–confirmed cases in 2020 by zip code.

The number of tele–urgent care visits doubled in 2020 (n=317) compared to 2019 (n=155). In addition, the distribution of the visits covered more geographic locations in North Carolina compared to the visits in 2019 that were primarily around the Durham-Raleigh area (Figure 3A and B). The northern and southern part of the North Carolina include zip codes with higher social flag scores, which indicates areas of higher social vulnerability (Figure 3C). The same regions with a high social flag score also experienced high rates of COVID-19–confirmed cases, which presents a challenge to an already vulnerable population (Figure 3D).

Table 2 shows that relatively more visits came from zip codes with lower social deprivation scores during the COVID-19 time period, even though the raw number of visits from *high* deprivation zip codes did increase. Using a Pearson chi-square test, we rejected the null hypothesis of independence between the COVID-19 time period and deprivation grouping at the 0.05 level with a *P* value of .04. Therefore, there was a significant association between the rate of COVID-19 cases and the concentration level of social factors in a given ZCTA.

Among 808 North Carolina zip codes, 181 (22%) consisted of a high concentration of vulnerable populations, where 17.7% (56/317) of the tele–urgent care visits originated from. The majority (215/317, 67.8%) of tele–urgent care visits originated from zip codes with a low concentration of vulnerable populations. Areas of high concentration of vulnerable populations experienced the highest rates of COVID-19 cases (214.8) and deaths (4.1) per 10,000.

**Table 2:** The use of tele–urgent care services among low, medium, and high regions of social vulnerability between March 1 and September 30, 2019 and 2020.

Social flag score	Zip codes, n	Telehealth visits Mar-Aug 2019, n (%)	Telehealth visits per 10,000 Mar-Aug 2019, n	Telehealth visits Mar-Aug 2020, n (%)	Telehealth visits per 10,000 Mar-Aug 2020, n	COVID-19–confirmed cases per 10,000, n	COVID-19 deaths per 10,000, n
Low (0-1)	417	93 (60)	0.71	215 (67.8)	1.64	138.2	2.0
Medium (2-3)	210	19 (12.3)	0.4	46 (14.5)	0.98	175.6	2.8
High (4-7)	181	43 (27.7)	0.9	56 (17.7)	1.18	214.8	4.1
Total	808	155	0.69	317	1.4	161.4	2.6

## Discussion

### Principal Findings

We conducted a cross-sectional study of pediatrics tele–urgent care visits before and after the COVID-19 pandemic among vulnerable populations. We found that the volume of tele–urgent care visits for pediatrics doubled after the pandemic when compared to the year before, which can be explained by the shutdown of health care systems during the initial phases of the COVID-19 pandemic. Post–COVID-19, there was a substantial shift in patient characteristics seeking tele–urgent care for pediatrics such that there were more women and patients with health insurance coverage compared to pre–COVID-19. Most of the postpandemic visits originated from the metropolitan Raleigh region. This could be explained by several reasons including the shutdown of in-person nonessential visits during the initial phases of the pandemic. In addition, there were strict COVID-19 social gathering restrictions, and many schools transitioned to virtual classrooms.

Post–COVID-19, we reported an overall substantial increase in pediatric tele–urgent care visits. Regions characterized with high poverty and high concentration of African American people encountered the highest increase in visits among all examined social factors. The high demand among these communities demonstrates high disparities for tele–urgent care use for pediatrics care during the pandemic. We recommend future exploration of current infrastructure and culture to adopting tele–urgent care such that patients living in areas of high poverty may not have access to tele–urgent care equipment or internet access, which may hinder the adoption of tele–urgent care among vulnerable populations. Therefore, offering video and telephone visit options to vulnerable populations may improve adoption and use levels of tele–urgent care.

Telehealth provides a convenient delivery method of health care for pediatric patients; however, the impact of tele–urgent care on patient outcomes remains unknown. We recommend future investigation of the effect of tele–urgent care on emergency department visits and urgent care clinic visits. Moreover, prescription rates were found to be different between video and telephone visits [16]. We recommend more investigations around medication prescription rates in tele–urgent care visits compared to in-person visits for pediatric care.

There appears to be a relationship between social flags and change in tele–urgent care visits between 2019 and 2020. Although a sizeable minority of tele–urgent care visits occurred in regions with high concentrations of vulnerable populations, the majority of tele–urgent care visits occurred in zip codes with low concentrations of vulnerable populations. Post–COVID-19, the volume of tele–urgent care visits in regions with a high concentration of vulnerable populations was less when compared to pre–COVID-19. Although the reason for such a drop in visits is unclear, it is possible that loss of employment and new local tele–urgent care clinics may have attributed to the decrease in visits. In addition, it is possible that during the pandemic newly established tele–urgent care services available through local clinics and primary care providers were preferred by patients.

Post–COVID-19, there were two spikes in the volume of tele–urgent care visits in March and July 2020 among most vulnerable population groups compared to two spikes in 2019 in March and April. The post–COVID-19 increase may be associated with the sudden shutdown of in-person appointments across the state in March 2020 in response to the World Health Organization announcing COVID-19 as a global pandemic [17]. Another explanation to the spikes in the month of March could be associated with the influenza and allergy seasons, which may explain the spike in both 2019 and 2020. In addition, the end of the school year coupled with the national holiday the Fourth of July may have attributed to the increase in visit volume during the month of July. Telehealth can be a suitable intervention to manage chronic care conditions within pediatric patients who may lack access during a pandemic due to the shutdown of schools and clinics [18].

Telehealth use in pediatric care has shown major increase in use during the pandemic. In the future, telehealth may be a suitable health care delivery modality that complements in-office pediatric visits for established patients. Although telehealth use has increased during the pandemic, there remains unanswered questions around the effectiveness of telehealth in pediatric care and the quality of care [19]. We recommend the integration of geospatial technologies to evaluate access factors such as broadband access, clinical effectiveness, medication prescription rates, and the acceptance of telehealth among pediatric patients and providers.

### Limitations

This study has several limitations. Although the tele–urgent care data is statewide, it represents a single virtual care center. The patient demographic form did not include ethnic or racial information, which limited our ability to map patient-level ethnic data to zip code–level ethnic data. In the future, patient demographic forms will include ethnic and racial fields. The comparison of 2019 and 2020 data may include confounding factors including the effect of marketing campaigns and word of mouth in increasing the volume of tele–urgent care visits.

### Conclusion

The use of tele–urgent care visits for pediatric care doubled during the COVID-19 pandemic. The majority of the tele–urgent care visits after COVID-19 originated from regions where there is a low presence of vulnerable populations. In addition, our geospatial analysis found that geographic regions with a high concentration of vulnerable populations had a significantly higher rate of COVID-19–confirmed cases and deaths compared to regions with a low concentration of vulnerable populations.

## Abbreviations

ACS: American Community Survey
SNAP: Supplemental Nutrition Assistance Program
ZCTA: Zip Code Tabulation Area
